# Enteropathogens and Gut Inflammation in Asymptomatic Infants and Children in Different Environments in Southern India

**DOI:** 10.4269/ajtmh.17-0324

**Published:** 2017-12-11

**Authors:** Ira Praharaj, R. Revathy, Rini Bandyopadhyay, Blossom Benny, Mohammed Azharuddin KO, Jie Liu, Eric R. Houpt, Gagandeep Kang

**Affiliations:** 1 Wellcome Trust Research Laboratory, Division of Gastrointestinal Sciences, Christian Medical College, Vellore, Tamil Nadu, India;; 2 Division of Infectious Diseases and International Health, University of Virginia, Charlottesville, Virginia

## Abstract

Children in poor environmental conditions are exposed early and often to enteric pathogens, but within developing countries, heterogeneity in enteropathogen exposure in different settings and communities is rarely addressed. We tested fecal samples from healthy infants and children from two different environments in the same Indian town for gut enteropathogens and biomarkers of gut inflammation. A significantly higher proportion of infants and children from a poor semi-urban neighborhood (93%) had one or more enteropathogens than those from a medical college campus (71.7%). Infants and children from the poor neighborhood had an average of 3.3 (95% confidence interval [CI]: 2.9–3.7) enteropathogens compared with an average of 1.4 (95% CI: 1.0–1.7) enteropathogens in campus infants/children. Viral and bacterial infections, including enteroviruses, adenoviruses, *Campylobacter* spp., and diarrhegenic *Escherichia coli* were more common and fecal biomarkers of inflammation were higher in the poor neighborhood. The findings demonstrate significant difference in the asymptomatic carriage of gut enteropathogens and gut inflammatory biomarkers in infants and children from two different environments within the same town in south India.

## INTRODUCTION

Environmental enteropathy (EE) or environmental enteric dysfunction (EED) is a poorly defined condition of the small intestine usually associated with gut inflammation and increased gut permeability. This condition is common in less developed settings and has been hypothesized to be linked to the high enteropathogen load in infants in developing countries, which leads to chronic enteric T-cell–mediated inflammation.^1^ This, in turn, has been linked with linear growth failure, impaired cognition, and suboptimal response to oral vaccines.^2^

Although studies, such as the “Etiology, Risk Factors and Interactions of Enteric Infections and Malnutrition and Consequences for Child Health and Development” (MAL-ED), have looked at biomarkers of EE among children in deprived settings, there are few studies evaluating differences, if any, in the levels of proposed biomarkers of EE or EED in children living in different environments in the same geographic location. The MAL-ED study looked at the role played by enteropathogen infection in malnutrition, gut inflammation, and other long-term health outcomes by focusing on resource-deprived settings with high incidence of diarrheal disease.^3^ However, this and other such studies have not evaluated heterogeneity in enteropathogen exposure and its consequences in communities with varying access to clean water and sanitation. In the following study, we compared the prevalence of asymptomatic enteropathogens and biomarkers of gut inflammation as a component of EE/EED,^4^ among infants and children living in different environmental conditions in southern India.

## MATERIALS AND METHODS

### Study populations.

Apparently healthy, asymptomatic infants and children aged 3 months to 4 years of age living in two different areas of Vellore in southern India were recruited between October 2014 and February 2015.

#### College campus.

Parents of infants and children living in the residential campus of the Christian Medical College (CMC), Vellore, were approached for stool specimens from their infants/children. Stool sample collection kits and detailed instructions, to collect samples only if no one in the household had diarrhea or use of antibiotics currently or in the previous week, were provided to all households whose members expressed interest in the study. Apart from the age and sex of the child, no other information was sought. The campus has good housing, adequate drinking water supply, closed drainage, and an absence of domestic animals other than pets. Water supply to the households on the campuses is chlorinated at source using a continuous chlorination disinfection system and continuous on-line chlorine monitoring is also performed to ensure proper disinfection. Drinking water samples are regularly tested for fecal coliform counts.

#### Chinnallapuram.

Stool samples were collected from asymptomatic infants/children living in a semi-urban area, Chinnallapuram, which is one of four adjacent semi-urban partly formal settlements (formerly described as slums), measuring 0.41 km^2^, located on the western outskirts of Vellore town with a population of approximately 50,000. There is intermittent supply (at intervals ranging from 2 to 14 days) of piped drinking water, resulting in home storage for extended periods. In previous studies carried out in this setting, the municipal water supply was found to be contaminated and the water storage practices have been found to increase contamination.^5^ In an earlier study in this area, 85% of all water samples tested positive for fecal coliforms.^6^ Although most of the households in this area have access to toilets, the final disposal of fecal waste or other solid waste is not ideal. The area has open drains which are cleaned by municipality workers. A proportion (18%) of households have domesticated cattle residing in or near the house.^6^

The Chinnallapuram area is comparable with the MAL-ED field site^7^ which is also situated in Vellore town, with regard to the access to clean drinking water and waste disposal practices. However, the rates of open defecation are much higher in the MAL-ED field site compared with the Chinnallapuram area in which the study being described is based.

The study was approved by the Institutional Review Board of CMC, Vellore.

### Enteropathogen detection and quantitation of fecal biomarkers.

Stool samples were received at room temperature from the campus and field staff, aliquoted and stored at −70°C before testing. This included aliquots with protease inhibitors. Total nucleic acid extraction was performed on all stool samples and custom-designed Taqman array cards for enteropathogens were used to detect enteric pathogen targets.^8^ The enteropathogen targets evaluated included enteric viruses, bacterial enteropathogens, and parasites and helminthes, as shown in Table 1 in supplementary materials. A Ct value of 35 cycles was used as the cutoff for all targets.

**Table 1:** Enteropathogens in infants/children of different age groups in two different environments in India

	3–6 months		6–12 months		1–4 years	
	Chinnallapuram	Hospital campus	Chinnallapuram	Hospital campus	Chinnallapuram	Hospital campus
Enteric viruses (% prevalence)	44.4	57.1*	80	14.3	70.3	12.0
Enterovirus	22.2	42.9*	47.5	0	51.4	12.8
Adenovirus	11.1	0	50	14.3	27.0	15.4
Rotavirus	11.1	14.3	7.5	14.3	0	2.6
Sapovirus	11.1	0	17.5	0	2.7	0
Norovirus GII	11.1	0	5.0	0	0	7.7
Bacterial enteropathogens (% prevalence)	55.6	14.3	95	85.7	81.1	64.1
*Campylobacter* spp.	14.3	0	35	0	21.6	5.1
EAEC	57.1	0	87.5	28.6	45.9	33.3
EPEC	57.1	11.1	45	42.9	40.5	33.3
ETEC	0	0	17.5	0	16.2	10.3
EIEC	14.3	0	7.5	0	2.7	5.1
*Salmonella* spp.	0	0	7.5	0	5.4	0
Enteric parasites (% prevalence)	0	0	20	0	48.6	0
*Giardia intestinalis*	0	0	7.5	0	45.9	0
*Cryptosporidium* spp.	0	0	7.5	0	5.4	0

EAEC = enteroaggregative *Escherichia coli*; EPEC = enteropathogenic *E. coli*; ETEC = enterotoxigenic *E. coli*; EIEC = enteroinvasive *E. coli*. This table includes only the most common pathogens (> 5% overall prevalence) in the pathogen groups (viruses, bacteria, and parasites).

* N.B. A majority (75%) of the enterovirus-positive samples from the hospital campus infants were found to be shedding Sabin vaccine polioviruses.

Fecal calprotectin and myeloperoxidase (MPO), both biomarkers of gut inflammation, were measured using commercial enzyme-linked immunosorbent assay kits (Hycult Biotech, the Netherlands) following manufacturer’s instructions. Fecal calprotectin levels were expressed in µg/g of feces, whereas MPO levels were expressed in ng/mL.

All statistical analysis was performed using Microsoft Excel, SPSS 17.0 and GraphPad Prism version 4. Two-tailed levels of significance were used and *P* < 0.05 was considered significant.

## RESULTS

From campus children, 53 stool samples in the age group 3 months to 4 years were obtained, whereas 86 samples were obtained from infants and children in Chinnallapuram in the same age group.

### Enteropathogens.

**Figure 1.**
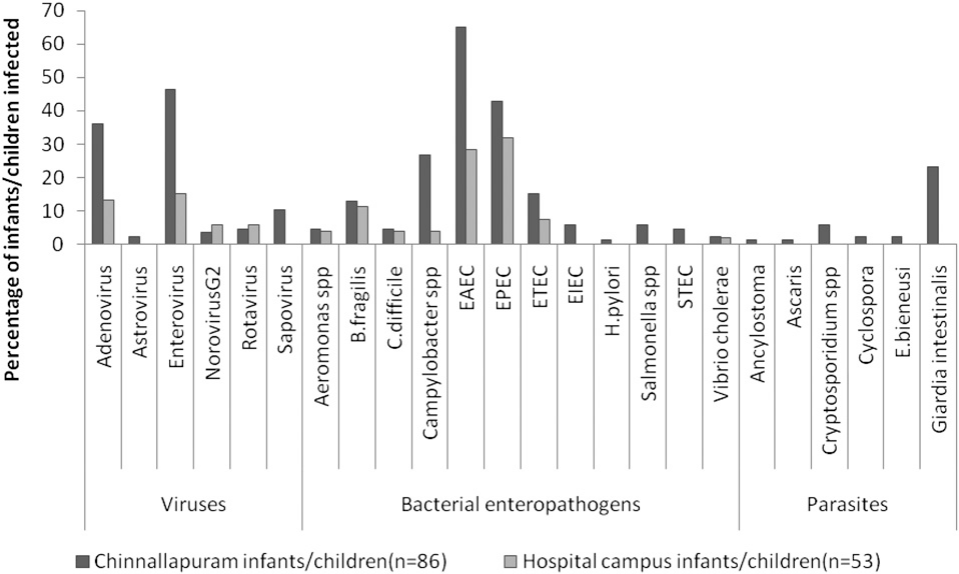
Gut enteropathogens in infants and children living in Chinnallapuram (a semi-urban poor neighborhood) and a medical college campus in Vellore, India.

A total of 38/53 (71.7%) and 80/86 (93%) of the campus and Chinnallapuram children, respectively, had at least one enteropathogen (χ^2^ test *P* value < 0.001). Children from Chinnallapuram had an average of 3.3 pathogens detected (95% confidence interval [CI]: 2.9–3.7, median 5, range 0–9), whereas campus infants/children had an average of 1.4 (95% CI: 1.0–1.7, median 1, range 0–4) pathogens detected using Taqman array card (TAC) assays (Mann–Whitney *U* test, *P* < 0.001). Figure 1 presents the enteropathogen profile in the two locations.

Children from Chinnallapuram had more viral (72.1%, 62/86 versus 32.1%, 17/53, *P* < 0.001), bacterial (84.9%, 73/86 versus 60.4%, 32/53, *P* = 0.001), and parasitic (30.2% 27/86 versus 0) pathogens than campus children. Enteric virus infections were significantly more common in children in the poor urban environment than in the campus (odds ratio [OR] = 9.2, 95% CI: 3.9–23.1).The difference was evident in the age group 6–12 months and 1–4 year age group children and was driven mostly by higher prevalence of enterovirus and adenovirus infections in these age groups (Table1).

Enteroviruses were the most common viral enteropathogens (46.5% Chinnallapuram, 15.1% campus), followed by adenovirus. Asymptomatic rotavirus infections did not differ significantly between infants/children in the two locations (4.6% Chinnallapuram, 5.7% campus). Among the bacterial enteropathogens, enteroaggregative *Escherichia coli* (EAEC), enteropathogenic *E. coli* (EPEC), enterotoxigenic *E. coli* (ETEC), and *Campylobacter* species predominated in both groups, (Figure 1). EAEC (68.5% versus 28.3%, *P* < 0.001) and *Campylobacter* species (26.4% versus 3.8%, *P* < 0.001) were significantly higher in Chinnallapuram compared with the campus residents. Among children tested from Chinnallapuram, the commonest enteric parasites were *Giardia intestinalis* (23.3%) and *Cryptosporidium* spp. (5.8%), whereas none of the infants/children residing in the campus were positive for any enteric parasites.

### Fecal biomarkers of gut inflammation.

**Figure 2.**
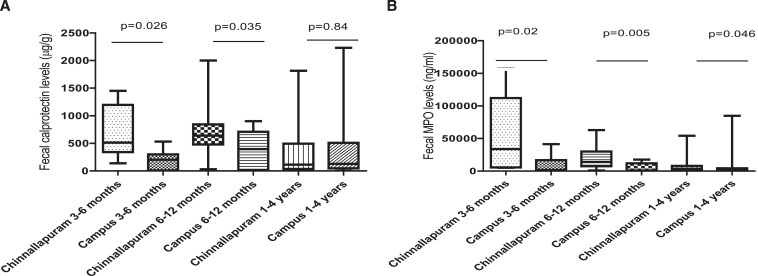
Fecal inflammatory biomarker levels in infants and children of different age groups living in Chinnallapuram (a semi-urban poor neighborhood) and a medical college campus in Vellore, India. (**A**) Fecal calprotectin levels in infants and children of different age groups living in Chinnallapuram (a semi-urban poor neighborhood) and a medical college campus. (**B**) Fecal myeloperoxidase (MPO) levels in infants and children of different age groups living in Chinnallapuram (a semi-urban poor neighborhood) and a medical college campus.

Fecal calprotectin and MPO were significantly higher in infants from Chinnallapuram compared with campus infants (Table 2). However, fecal calprotectin levels did not differ significantly in the two settings among children 1–4 years of age (Figure 2). In an age-adjusted analysis, the difference in the levels of fecal calprotectin between infants from the two settings was statistically significant in infants 3–6 months (Mann–Whitney *U* test, *P* = 0.02) and 6–12 months (Mann–Whitney *U* test, *P* = 0.035). Fecal MPO levels were significantly higher in Chinnallapuram infants/children compared with hospital campus–dwelling infants and children in all the age groups analyzed. Correlation between fecal calprotectin and MPO levels was high (Spearman’s *r* = 0.84, 95% CI: 0.79–0.88, *P* < 0.0001).

**Table 2:** Levels of fecal calprotectin and myeloperoxidase (MPO) in infants/children residing in a medical college campus compared with those in a poor semi-urban neighborhood (Chinnallapuram)

	Hospital campus infants/children	Chinnallapuram infants/children	Two-tailed *P* value (Mann–Whitney U test)
*N*	53	83*	–
Fecal calprotectin (µg/g)			
Median (IQR)	130 (38–490)	492 (140–760)	0.0004
Mean	310	570	–
Fecal MPO (ng/mL)			
Median (IQR)	1,080 (490–4,300)	8,095 (2,400–22,000)	–
Mean	6,460	18,086	< 0.0001

IQR = interquartile range.

* Inflammatory biomarker testing results were available for *N* = 83 of 86 infants/children from Chinnallapuram for whom enteropathogen testing was performed.

### Association between fecal biomarkers of gut inflammation and enteropathogens.

Table 3 presents the fecal calprotectin and MPO levels in samples having different types of gut enteropathogens. Both fecal calprotectin and MPO levels were significantly higher in samples where multiple enteropathogens were detected compared with those with single or no enteropathogens (Kruskal–Wallis Test, *P* < 0.0001). Although generally, samples with bacterial enteropathogens had higher levels of fecal calprotectin and MPO compared with those positive for enteric viruses and parasites (Table 3), this difference was not statistically significant.

**Table 3:** Fecal calprotectin and myeloperoxidase (MPO) levels in samples with different types of enteropathogens

	No enteropathogens	Any enteropathogen	Single enteropathogen	Multiple enteropathogens	Only bacterial enteropathogens	Only enteric viruses
*N*	21	115	32	83	33	12
Fecal calprotectin (µg/g)						
Median (IQR)	133 (20–679)	341 (99–737)	134 (48–393)	497 (431–767)	189 (51–193)	129 (40–219)
Fecal MPO (ng/mL)						
Median (IQR)	1,073 (552, 7,963)	5,152 (1,100, 17,829)	1,378 (565, 5,140)	8,095 (1,993, 22,252)	2,611 (701, 9,771)	1,977 (466, 5,431)

IQR = interquartile range.

## DISCUSSION

Enteropathogens and fecal biomarkers of inflammation were lower in children living in better environmental conditions in southern India. In a study by Taniuchi et al.^9^ asymptomatic infants residing in Bangladesh had much higher enteropathogen load compared with infants from the United States, but there are no previous reports comparing enteropathogens and fecal biomarkers in asymptomatic children in different environmental conditions in the same geographic location. A study from a peri-urban region in Peru evaluated enteropathogen load in low socioeconomic settings and evaluated associations of enteropathogen positivity in diarrhea with socioeconomic status (SES) and other variables considered to be “SES indicators” such as mode of water storage. The study concluded that although SES is not independently associated with diarrheal incidence, incidence does correlate with transmission factors such as water storage conditions and geophagy in infants, which are associated with SES.^10^ Liu et al.^11^ reported higher fecal calprotectin levels in rural infants compared with infants in urban settings in China.

The presence of gut pathogens in apparently healthy children has been referred to as the gut “pathobiome”^12^ and has been hypothesized to be causal in the development of EE. Asymptomatic presence of enteropathogens and a state of chronic gut inflammation has been hypothesized to be associated with a host of phenomenon ranging from growth retardation, impairment of cognitive function, and decreased responses to oral vaccines.^13^

In our study, enteric virus infections were more common among children in Chinnallapuram compared with children residing on the campus. This difference was driven by significantly lower prevalence of enteroviruses and adenovirus infections among the campus children in the 6- to 12-month and 1- to 4-year age groups. The proportion of children infected with enteroviruses and adenoviruses were significantly higher in the older age groups (6–12 months and 1–4 years) compared with infants less than 6 months of age. A dose-dependent protective effect of breast milk in the first 6 months of life has been reported for this age-related trend for enterovirus infection.^14^

In both settings, diarrheagenic *E.coli* and *Campylobacter* spp. were the most prevalent bacterial pathogens. Asymptomatic excretion may follow acute diarrhea, but may also be due to host immunity. After acute diarrhea, *Campylobacter jejuni/coli* can have prolonged shedding.^15^ Contact with domesticated animals and poultry is a potential exposure route for *Campylobacter jejuni*. Although presence of domesticated animals in household is a common feature of the urban setting in Vellore,^6^ for this study, we did not collect specific information on the presence of animals in the households.

None of the children from the hospital campus setting were positive for any enteric parasites, whereas the commonest enteric parasites among the slum-dwelling children were *G. intestinalis* and *Cryptosporidium* spp., both parasites with potential zoonotic routes of transmission which have been detected in drinking water sources such as ponds and shallow tubewell settings in India with considerable presence of cattle and other domesticated animals.^16^

High rates of enteropathogen positivity in asymptomatic children have been highlighted by two major studies on diarrheal etiology in recent days— the Global Enteric Multisite Study (GEMS study) and the MAL-ED multisite birth cohort study on enteric infections and malnutrition. In the GEMS case–control study, 72% of all healthy controls harbored one or more enteric pathogens.^17^ Similarly, 64.9% of all non-diarrheal surveillance samples from the MAL-ED study were positive for at least one putative enteropathogen using conventional microbiology methods, such as stool culture, antigen detection, and microscopy.^18^ In comparison, our study which used a more sensitive quantitative polymerase chain reaction–based method found 93% of all infants/children evaluated in the semi-urban low income setting to harbor one or more enteropathogens. Similar to our study, the commonest enteric pathogens in non-diarrheal samples in the MAL-ED study were diarrheagenic *E. coli* and *Campylobacter* species.

Inflammatory biomarkers such as MPO and calprotectin have been found to have a strong age-effect, with levels stabilizing after 2 years of age,^19^ and age-adjusted analysis showed that although the levels in both Indian settings evaluated here were much higher than in infants in developed countries,^20^ there was a significant difference between the semi-urban and campus infants. However, in 1- to 4-year old children, this difference by setting did not persist for fecal calprotectin levels. As stated earlier, one of the few studies where gut inflammatory biomarker levels of infants under different environmental conditions have been assessed is a cross-sectional comparison of fecal calprotectin levels among Chinese infants in rural and urban settings which showed much higher levels of fecal calprotectin among the rural infants.^11^ Fecal MPO was evaluated in the MAL-ED cohort study as a marker of gut inflammation and levels of this biomarker in asymptomatic children in the first 2 years of life were assessed.^4^ Similar to our study, the levels were significantly higher than published levels from high-income countries.^19^ The study also reported higher fecal MPO levels to be associated with lower WAMI scores.

Our study had several limitations. The numbers of children evaluated are small, place of residence was used as indicator of socioeconomic status without specific classification of each household, and there was no objective assessment of the environment or hygiene behavior of each household. However, we have previously assessed the socioeconomic status and household hygiene levels in Chinnallapuram and have found most families classified as low-middle or low-income status by the modified Kuppuswamy scale,^21^ whereas all campus residents are of high socioeconomic status by the same scale. Another potential limitation of this study is that the assessment of fecal biomarkers is confined to only fecal calprotectin and MPO, both of which are only indicative of gut inflammation and not intestinal permeability.

This study demonstrates the heterogeneity in the gut environment in infants and children in a single town in India. This is important because understanding heterogeneity permits assessment of the potential for improvement and has implications for diarrheal disease, EE, and potentially for the performance of oral vaccines.

## Supplementary Material

Supplemental Table.
