# Supplementary material for: Enteropathogens and Gut Inflammation in Asymptomatic Infants and Children in Different Environments in Southern India
Source: Am J Trop Med Hyg. 2017 Dec 11;98(2):576–80. doi: 10.4269/ajtmh.17-0324 (PMC5929183; doi:10.4269/ajtmh.17-0324)
Supplement: Supplementary file 1 [file tpmd170324.SD1.pdf]

Supplemental Table 1

Enteropathogen Taqman array card targets

Target	Gene	Reference
Bacterial targets		
Bacterial 16S	16S	22
Aeromonas	Aerolysin	8
Bacteroidesfragilis	EGBF	Modified from 23
Campylobacter	Cpn60	Designed based on 24
Campylobacter jejuni/coli	cadF	25
Clostridium difficile	tcdB	8
EAEC	aaiC	26
EAEC	aatA	26
EPEC	Eae	8
EPEC	bfpA	8
ETEC	LT	27
ETEC	ST	8
Helicobacter pylori	ureC	Designed based on 28
Mycobacterium tuberculosis	IS6110	29
Salmonella	Ttr	30
Shigella/EIEC	ipaH	31
STEC	stx1	32
STEC	stx2	27
Vibrio cholerae	lyA	8
Eukaryotic targets		
Ancylostoma	Ribosomal gene	32
Ascaris	Ribosomal gene	Modified from 33
Cryptosporidium	Ribosomal gene	8
Cryptosporidium typing	Lib13	34
Cyclospora	Ribosomal gene	35
Enterocytozoonbieneusi	Ribosomal gene	36
Entamoeba histolytica	18S	37
Encephalitozoon intestinalis	Ribosomal gene	36
Giardia	18S	37
Giardia typing	tpi	Modified from 38
Isospora	Ribosomal gene	39
Necator	Ribosomal gene	32
Strongyloides	Dispersed repetitive sequence	40
Trichuris	Ribosomal gene	8
Viral targets		
Adenovirus serotypes 40/41	Fiber gene	41
Adenovirus	Hexon	42
Astrovirus	Capsid	8
Enterovirus	5′ UTR	Modified from 43
Norovirus genogroup GI	ORF1-2	Modified from 44
Norovirus genogroup GII	ORF1-2	44
Rotavirus	NSP3	45
Sapovirus	RdRp	8

EAEC = enteroaggregative *Escherichia coli*; EIEC = enteroinvasive *E. coli*; EPEC = enteropathogenic *E. coli*; ETEC = enterotoxigenic *E. coli*; LT = heat-labile enterotoxin; PhHV = phocine herpesvirus; ST = heat-stable enterotoxin; STEC = shiga toxin–producing *E. coli*.

SUPPLEMENTAL REFERENCES

22. Rousselon N, Delgenes JP, Godon JJ, 2004. A new real time PCR (TaqMan PCR) system for detection of the16S rDNA gene associated with fecal bacteria. *J Microbiol Methods 59:* 15–22.
23. Merino VR, Nakano V, Liu C, Song Y, Finegold SM, Avila-Campos MJ, 2011. Quantitative detection of enterotoxigenic *Bacteroides fragilis* subtypes isolated from children with and without diarrhea. *J Clin Microbiol 49:* 416–418.
24. Hill JE, Paccagnella A, Law K, Melito PL, Woodward DL, Price L, Leung AH, Ng LK, Hemmingsen SM, Goh SH, 2006. Identification of *Campylobacter* spp. and discrimination from *Helicobacter* and *Arcobacter* spp. by direct sequencing of PCR-amplified cpn60 sequences and comparison to cpnDB, a chaperonin reference sequence database. *J Med Microbiol 55:* 393–399.
25. Cunningham SA, Sloan LM, Nyre LM, Vetter EA, Mandrekar J, Patel R, 2010. Three-hour molecular detection of *Campylobacter*, *Salmonella*, *Yersinia*, and *Shigella* species in feces with accuracy as high as that of culture. *J Clin Microbiol 48:* 2929–2933.
26. Boisen N, Struve C, Scheutz F, Krogfelt KA, Nataro JP, 2008. New adhesin of enteroaggregative *Escherichia coli* related to the Afa/Dr/AAF family. *Infect Immun 76:* 3281–3292.
27. Hidaka A, Hokyo T, Arikawa K, Fujihara S, Ogasawara J, Hase A, Hara-Kudo Y, Nishikawa Y, 2009. Multiplex real-time PCR for exhaustive detection of diarrhoeagenic *Escherichia coli*. *J Appl Microbiol 106:* 410–420.
28. Shukla SK, Prasad KN, Tripathi A, Ghoshal UC, Krishnani N, Nuzhat H, 2011. Quantitation of *Helicobacter pylori* ureC gene and its comparison with different diagnostic techniques and gastric histopathology. *J Microbiol Methods 86:* 231–237.
29. Halse TA, Edwards J, Cunningham PL, Wolfgang WJ, Dumas NB, Escuyer VE, Musser KA, 2010. Combined real-time PCR and rpoB gene pyrosequencing for rapid identification of *Mycobacterium tuberculosis* and determination of rifampin resistance directly in clinical specimens. *J Clin Microbiol 48:* 1182–1188.

30. Malorny B, Paccassoni E, Fach P, Bunge C, Martin A, Helmuth R, 2004. Diagnostic real-time PCR for detection of *Salmonella* in food, *Appl Environ Microbiol 70:* 7046–7052.
31. Vu DT et al., 2004. Detection of Shigella by a PCR assay targeting the ipaH gene suggests increased prevalence of shigellosis in Nha Trang, Vietnam. *J Clin Microbiol 42:* 2031–2035.
32. Basuni M, Muhi J, Othman N, Verweij JJ, Ahmad M, Miswan N, Rahumatullah A, Aziz FA, Zainudin NS, Noordin R, 2011. A pentaplex real-time polymerase chain reaction assay for detection of four species of soil-transmitted helminths. *Am J Trop Med Hyg 84:* 338–343.
33. Wiria AE et al., 2010. Does treatment of intestinal helminth infections influence malaria? Background and methodology of a longitudinal study of clinical, parasitological and immunological parameters in Nangapanda, Flores, Indonesia (ImmunoSPIN Study). *BMC Infect Dis 10:* 77.
34. Hadfield SJ, Robinson G, Elwin K, Chalmers RM, 2011. Detection and differentiation of *Cryptosporidium* spp. in human clinical samples by use of real-time PCR. *J Clin Microbiol 49:* 918–924.
35. Verweij JJ, Laeijendecker D, Brienen EA, van Lieshout L, Polderman AM, 2003. Detection of *Cyclospora cayetanensis* in travellers returning from the tropics and subtropics using microscopy and real-time PCR. *Int J Med Microbiol 293:* 199–202.
36. Verweij JJ, Ten Hove R, Brienen EA, van Lieshout L, 2007. Multiplex detection of *Enterocytozoon bieneusi* and *Encephalitozoon* spp. in fecal samples using real-time PCR. *Diagn Microbiol Infect Dis 57:* 163–167.
37. Verweij JJ, Blange RA, Templeton K, Schinkel J, Brienen EA, van Rooyen MA, van Lieshout L, Polderman AM, 2004. Simultaneous detection of *Entamoeba histolytica*, *Giardia lamblia*, and *Cryptosporidium parvum* in fecal samples by using multiplex real-time PCR. *J Clin Microbiol 42:* 1220–1223.
38. Almeida A, Pozio E, Caccio SM, 2010. Genotyping of *Giardia duodenalis* cysts by new real-time PCR assays for detection of mixed infections in human samples. *Appl Environ Microbiol 76:* 1895–1901.
39. Ten Hove R, van Lieshout L, Brienen EA, Perez MA, Verweij JJ, 2008. Real-time polymerase chain reaction for detection of *Isospora belli* in stool samples. *Diagn Microbiol Infect Dis 61:* 280–283.
40. Verweij JJ, Canales M, Polaman K, Ziem J, Brienen EA, Polderman AM, van Lieshout L, 2009. Molecular diagnosis of *Strongyloides stercoralis* in faecal samples using real-time PCR. *Trans R Soc Trop Med Hyg 103:* 342–346.
41. Jothikumar N, Cromeans TL, Hill VR, Lu X, Sobsey MD, Erdman DD, 2005. Quantitative real-time PCR assays for detection of human adenoviruses and identification of serotypes 40 and 41. *Appl Environ Microbiol 71:* 3131–3136.
42. Heim A, Ebnet C, Harste G, Pring-Akerblom P, 2003. Rapid and quantitative detection of human adenovirus DNA by real-time PCR. *J Med Virol 70:* 228–239.
43. Oberste MS, Penaranda S, Rogers SL, Henderson E, Nix WA, 2010. Comparative evaluation of Taqman real-time PCR and semi-nested VP1 PCR for detection of enteroviruses in clinical specimens. *J Clin Virol 49:* 73–74.
44. Kageyama T, Kojima S, Shinohara M, Uchida K, Fukushi S, Hoshino FB, Takeda N, Katayama K, 2003. Broadly reactive and highly sensitive assay for Norwalk-like viruses based on real-time quantitative reverse transcription-PCR. *J Clin Microbiol 41:* 1548–1557.
45. Zeng SQ, Halkosalo A, Salminen M, Szakal ED, Puustinen L, Vesikari T, 2008. One-step quantitative RT-PCR for the detection of rotavirus in acute gastroenteritis. *J Virol Methods 153:* 238–240.
